# Supplementary material for: Reclassifying tumour cell cycle activity in terms of its tissue of origin
Source: NPJ Precis Oncol. 2022 Aug 20;6:59. doi: 10.1038/s41698-022-00302-7 (PMC9392789; doi:10.1038/s41698-022-00302-7)
Supplement: Supplementary file 1 — Supplementary material [file 41698_2022_302_MOESM1_ESM.pdf]

Supplementary Figure 1.

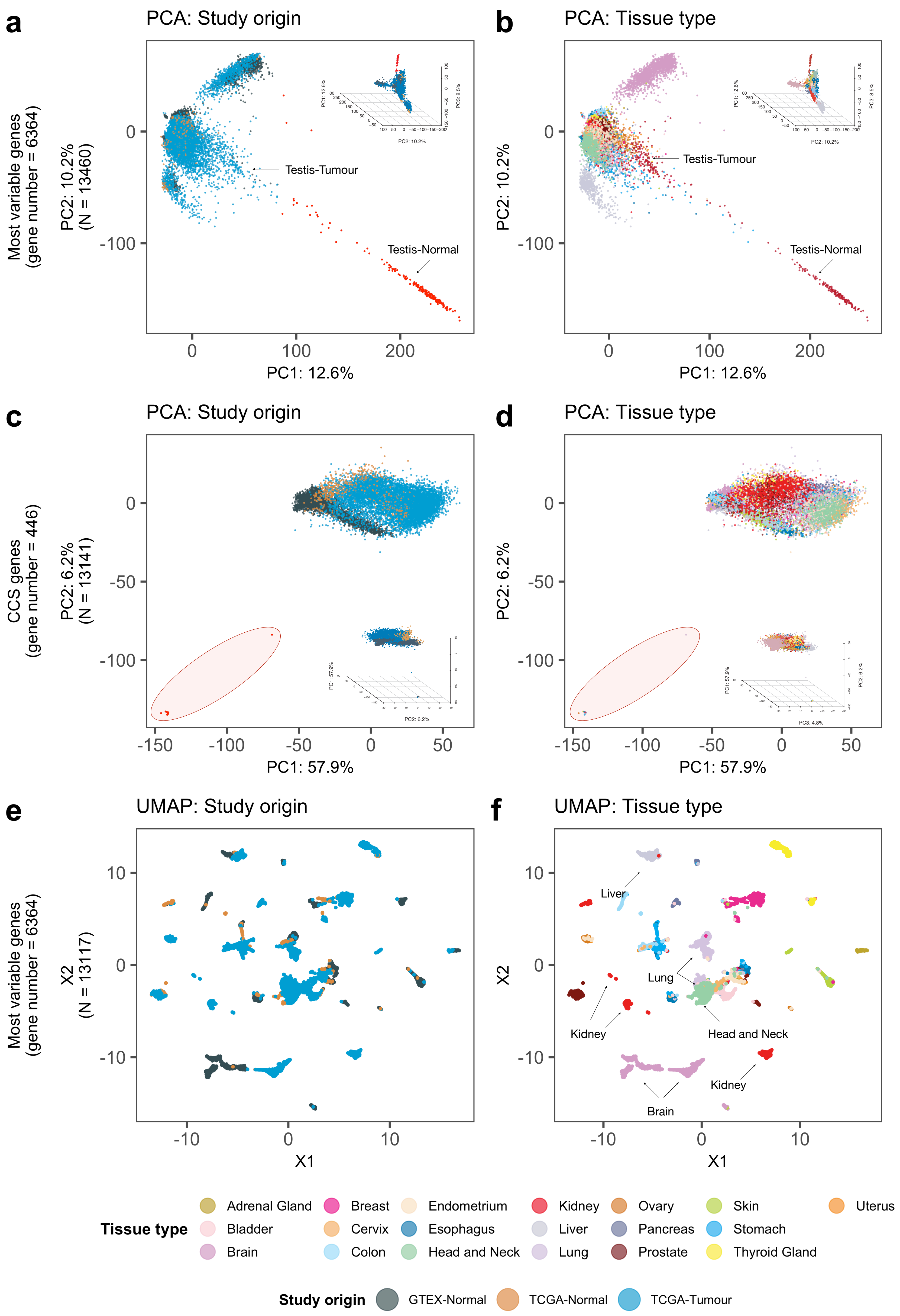

Supplementary Figure 1. Dimensionality reduction of Pan-Cancer and GTEx data
Principle component analyses (PCA) using most variable genes in the data: a) based on study origin and b) tissue types; PCA plots of the data using genes incorporated in the Cell cycle score (CCS): c) based on study origin and d) tissue types. Uniform Manifold Approximation and Projection (UMAP) plots of the data representing the clusters of tumors: based on e) Tissue type and f) Study origin after exclusion of the outliers.

Supplementary Figure 2.

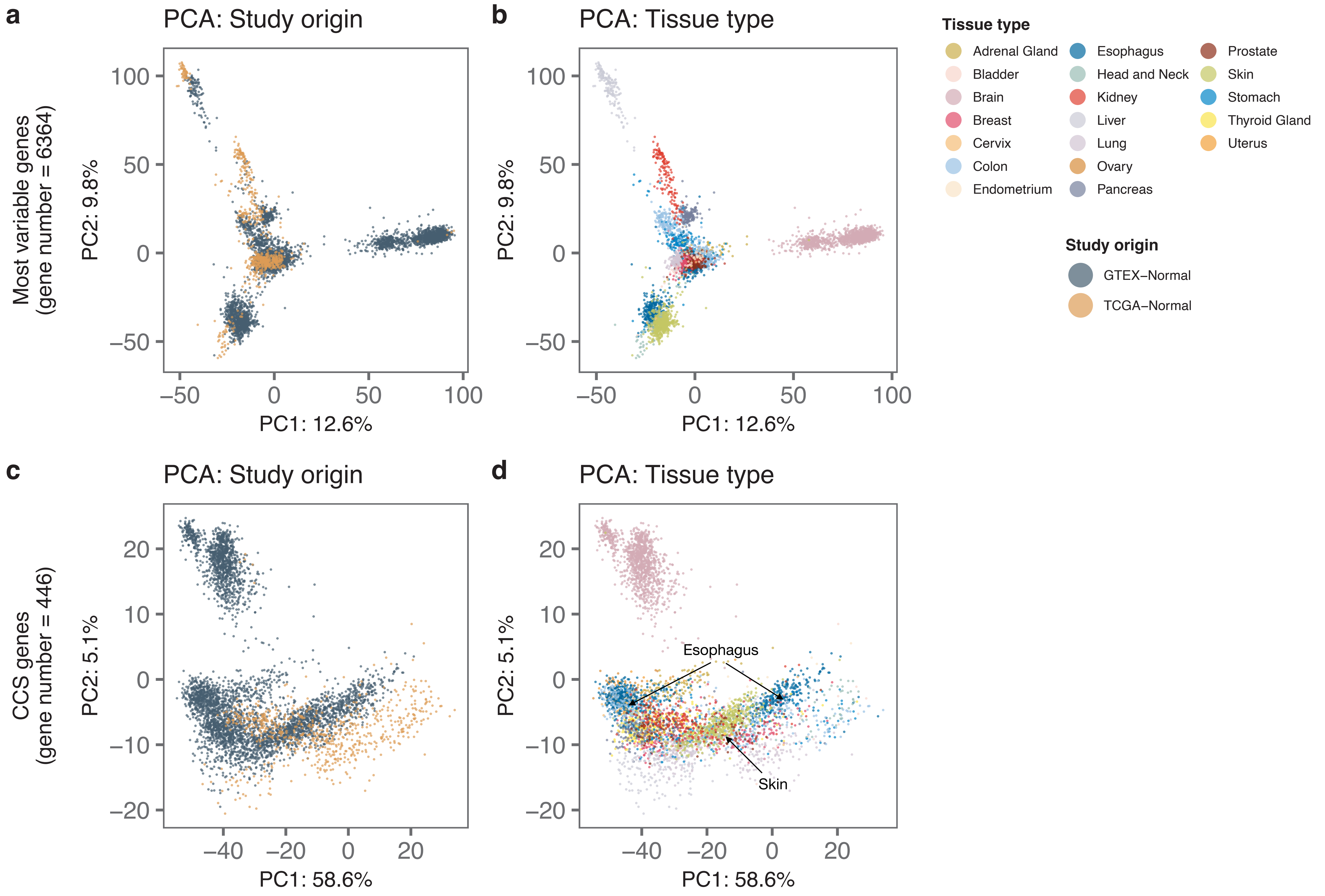

Supplementary Figure 2. Dimensionality reduction of normal samples from TCGA and GTEx data

Principle component analyses (PCA) using most variable genes in the data: a) based on study origin and b) tissue types; PCA plots of the data using genes incorporated in the Cell cycle score (CCS): c) based on study origin and d) tissue types.

Note that for some cancer types there are only GTEx normals or TCGA normals, all cancer types do not have normal samples from both studies.

Supplementary Figure 3.

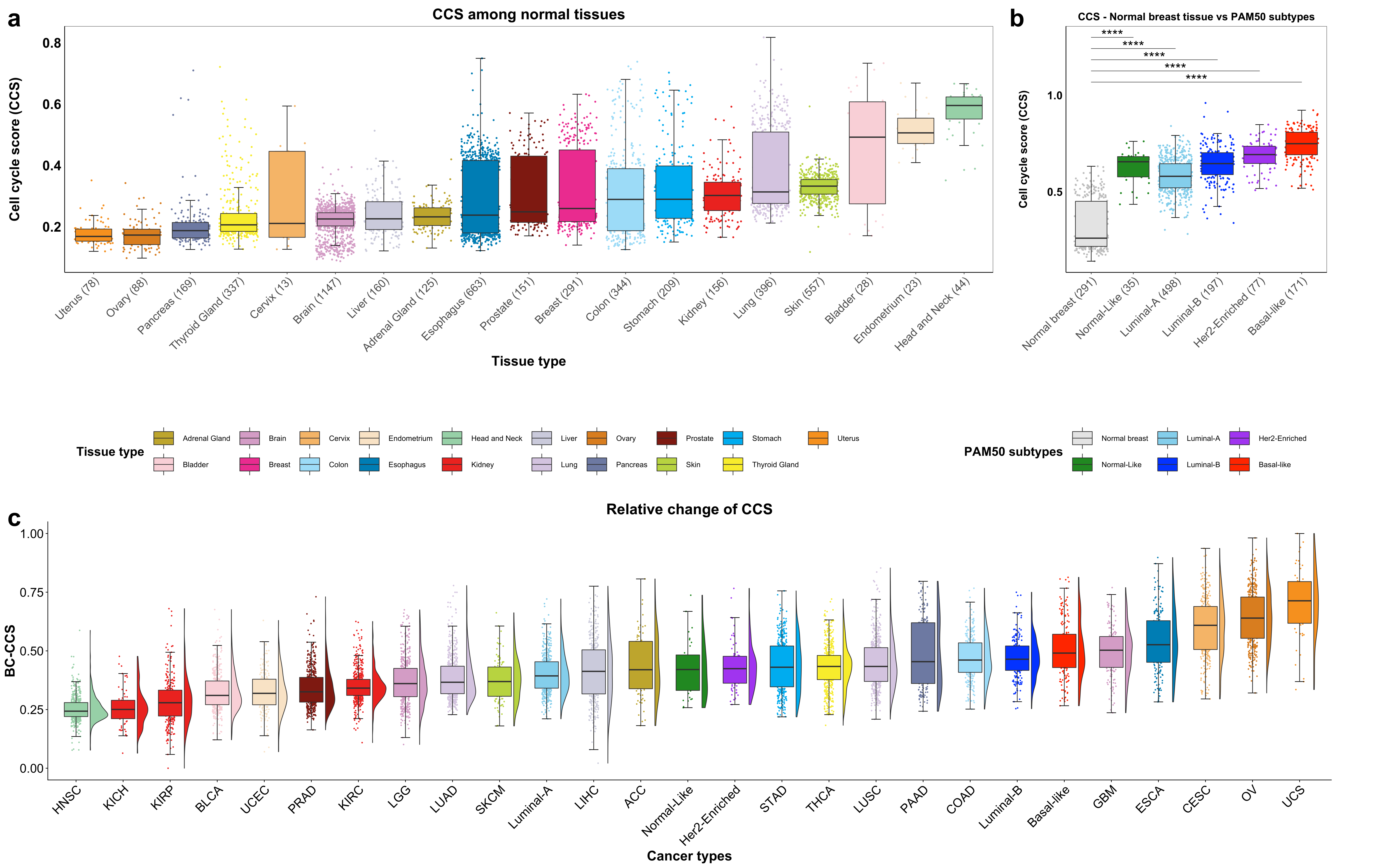

Supplementary Figure 3. Cell cycle activity among normal tissues and PAN-Cancer samples.
a) Boxplots representing Cell cycle score (CCS) as a surrogate for cell cycle activity among normal tissues. b) CCS score range among breast cancer PAM50 subtypes c) Boxplots and violin plots showing the Baseline Corrected - Cell Cycle Score (BC-CCS) among different tumour types including PAM50 subtypes of breast cancer tumours. Within each box, horizontal lines denote median values; boxes extend from the 25th to the 75th percentile of each group's distribution of values; vertical extending lines denote adjacent values (the most extreme values within 1.5 interquartile range of the 25th and 75th percentile of each group).

Supplementary Figure 4.

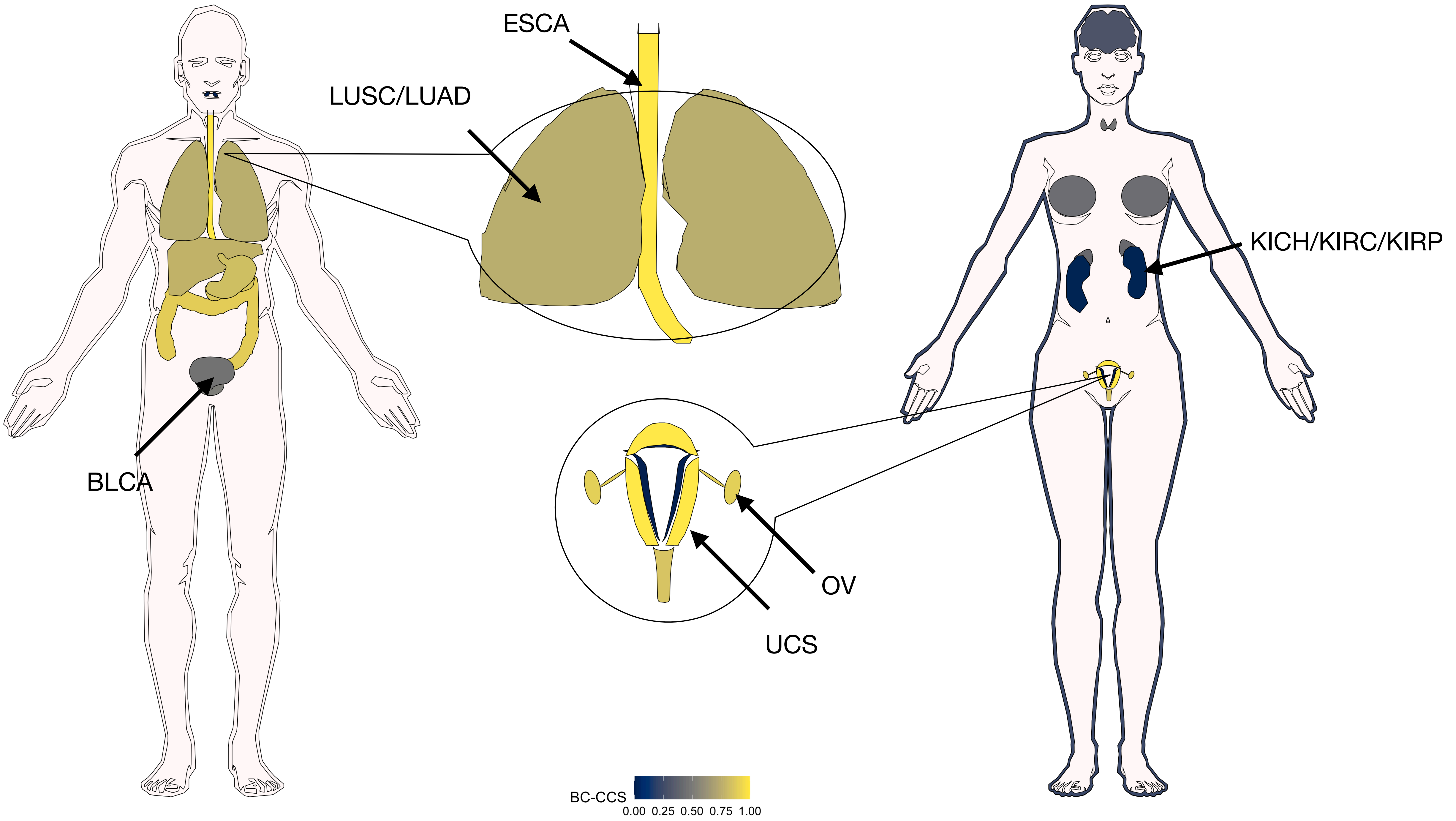

Supplementary Figure 4. Baseline Corrected - Cell Cycle Score (BC-CCS) at PAN-Cancer level

An anatomical visualization of BC-CCS among PAN-Cancer. Bladder Urothelial Carcinoma (BLCA), Esophageal carcinoma (ESCA), Kidney Chromophobe (KICH), Kidney renal clear cell carcinoma (KIRC), Kidney renal papillary cell carcinoma (KIRP), Lung adenocarcinoma (LUAD), Lung squamous cell carcinoma (LUSC), Ovarian serous cystuadenocarcinoma (OV) and Uterine Carcinosarcoma (UCS).

Supplementary Figure 5.

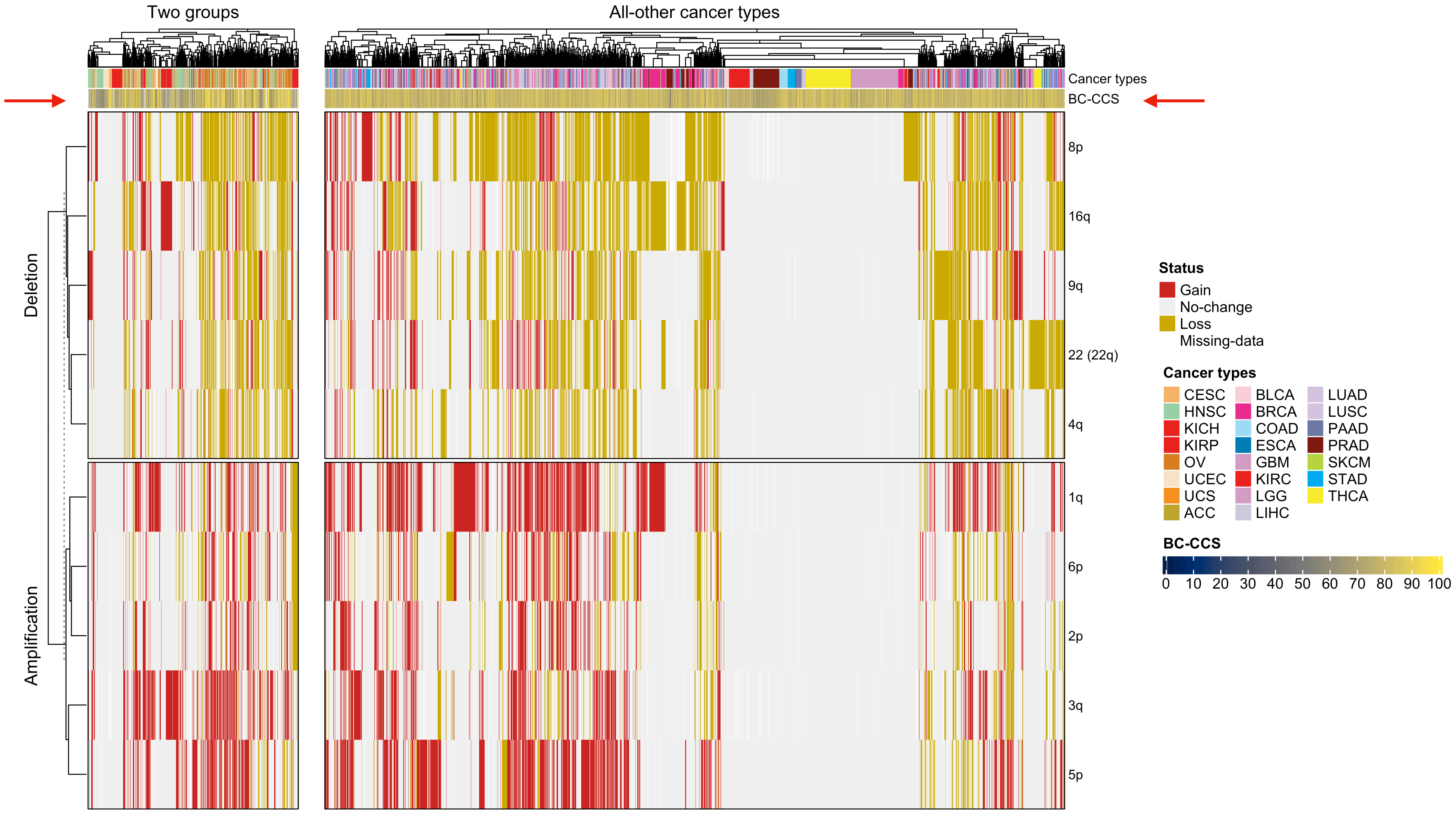

Supplementary Figure 5. Heatmap of aneuploidy status at PAN-Cancer level.
A heatmap representing the amplification and deletion status of tumors at top 5 chromosomal locations with highest Gain/Loss in Group 2 relative to Group 1 as shown in Figure 4D and E, respectively. BC-CCS: Baseline Corrected - Cell Cycle Score.

Supplementary Figure 6.

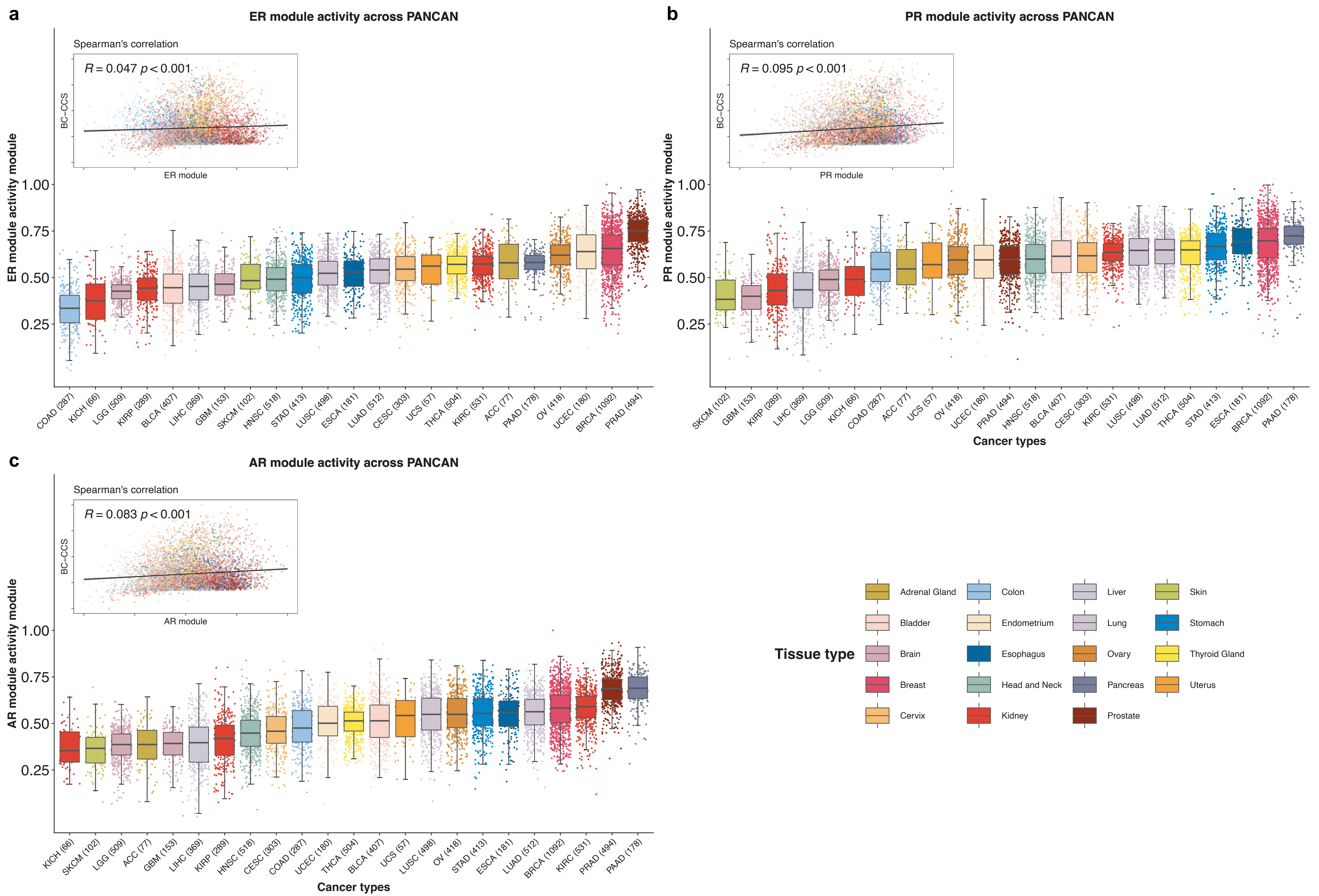

Supplementary Figure 6. Estrogen, Progesterone and Androgen gene modules activity across PANCAN

Box plots representing the a) Estrogen (ER), b) Progesterone (PR), c) Androgen (AR) gene modules activity across PANCAN. Spearman's correlation *R* shows the correlation between Baseline Corrected - Cell Cycle Score (BC-CCS) and ER,PR and AR modules. Within each box, horizontal lines denote median values; boxes extend from the 25th to the 75th percentile of each group's distribution of values; vertical extending lines denote adjacent values (the most extreme values within 1.5 interquartile range of the 25th and 75th percentile of each group).

Supplementary Table 1.
Number of outliers according to PCA

Tissue of origin	Variable genes		CCS	
	Normal	Tumour	Normal	Tumour
Adrenal Gland	0	0	1	0
Brain	0	0	6	0
Colon	0	0	4	0
Esophagus	0	0	4	0
Kidney	0	0	1	0
Lung	0	0	1	1
Ovary	0	0	0	1
Pancreas	0	0	2	0
Stomach	0	0	2	0
Thyroid Gland	0	0	1	0
Testis	156	163	0	0
Total	156	163	22	2
	319		24	

Supplementary Table 2.
Tumour purity level among different cancer types

Tissue of origin	Cancer type	Avg. purity
Adrenal Gland	ACC	0.83
Bladder	BLCA	0.62
Brain	LGG	0.72
Brain	GBM	0.76
Breast	BRCA	0.61
Cervix	CESC	0.66
Colon	COAD	0.64
Endometrium	UCEC	0.73
Esophagus	ESCA	0.63
Head and Neck	HNSC	0.52
Kidney	KIRC	0.58
Kidney	KIRP	0.74
Kidney	KICH	0.82
Liver	LIHC	0.7
Lung	LUAD	0.47
Lung	LUSC	0.52
Ovary	OV	0.78
Pancreas	PAAD	0.55
Prostate	PRAD	0.62
Skin	SKCM	0.68
Stomach	STAD	0.53
Thyroid Gland	THCA	0.72
Uterus	UCS	0.82

Avg. purity: Average tumour purity, ACC: Adrenocortical carcinoma, BLCA: Bladder Urothelial Carcinoma, BRCA: Breast invasive carcinoma, CESC: Cervical squamous cell carcinoma and endocervical adenocarcinoma, COAD: Colon adenocarcinoma, ESCA: Esophageal carcinoma, GBM: Glioblastoma multiforme, HNSC: Head and Neck squamous cell carcinoma, KICH: Kidney Chromophobe, KIRC: Kidney renal clear cell carcinoma, KIRP: Kidney renal papillary cell carcinoma, LGG: Brain Lower Grade Glioma, LIHC: Liver hepatocellular carcinoma, LUAD: Lung adenocarcinoma, LUSC: Lung squamous cell carcinoma, OV: Ovarian serous cystadenocarcinoma, PAAD: Pancreatic adenocarcinoma, PRAD: Prostate adenocarcinoma, SKCM: Skin Cutaneous Melanoma, STAD: Stomach adenocarcinoma, THCA: Thyroid carcinoma, THYM: Thymoma, UCS: Uterine Carcinosarcoma; tumour purity estimation was calculated by ABSOLUTE algorithm (ref no.11)
