# Supplementary material for: Reclassifying tumour cell cycle activity in terms of its tissue of origin
Source: NPJ Precis Oncol. 2022 Aug 20;6:59. doi: 10.1038/s41698-022-00302-7 (PMC9392789; doi:10.1038/s41698-022-00302-7)
Supplement: Supplementary file 3 — REPORTING SUMMARY [file 41698_2022_302_MOESM3_ESM.pdf]

nature portfolio

Corresponding author(s): Nick Tobin
Last updated by author(s): Jun 16, 2022

Reporting Summary

Nature Portfolio wishes to improve the reproducibility of the work that we publish. This form provides structure for consistency and transparency in reporting. For further information on Nature Portfolio policies, see our Editorial Policies and the Editorial Policy Checklist.

Statistics

For all statistical analyses, confirm that the following items are present in the figure legend, table legend, main text, or Methods section.

n/a	Confirmed	
☐	☒	The exact sample size (*n*) for each experimental group/condition, given as a discrete number and unit of measurement
☐	☒	A statement on whether measurements were taken from distinct samples or whether the same sample was measured repeatedly
☐	☒	The statistical test(s) used AND whether they are one- or two-sided<br>*Only common tests should be described solely by name; describe more complex techniques in the Methods section.*
☐	☒	A description of all covariates tested
☐	☒	A description of any assumptions or corrections, such as tests of normality and adjustment for multiple comparisons
☐	☒	A full description of the statistical parameters including central tendency (e.g. means) or other basic estimates (e.g. regression coefficient) AND variation (e.g. standard deviation) or associated estimates of uncertainty (e.g. confidence intervals)
☐	☒	For null hypothesis testing, the test statistic (e.g. *F*, *t*, *r*) with confidence intervals, effect sizes, degrees of freedom and *P* value noted<br>*Give P values as exact values whenever suitable.*
☒	☐	For Bayesian analysis, information on the choice of priors and Markov chain Monte Carlo settings
☒	☐	For hierarchical and complex designs, identification of the appropriate level for tests and full reporting of outcomes
☐	☒	Estimates of effect sizes (e.g. Cohen's *d*, Pearson's *r*), indicating how they were calculated

Our web collection on statistics for biologists contains articles on many of the points above.

Software and code

Policy information about availability of computer code

Data collection	The data was obtained from https://xenabrowser.net/datapages/?hub=https://toil.xenahubs.net:443<br>The annuploidy score (Aneuploidy scores and arm calls file - PANCAN_ArmCallsAndAneuploidyScore_092817.txt): https://gdc.cancer.gov/about-data/publications/pancan-aneuploidy<br>The tumour purity data: https://gdc.cancer.gov/about-data/publications/pancanatlas<br>also PAM50 subtypes: retrieved from cBioportal: http://www.cbioportal.org "PanCanacer Studies data"
Data analysis	All custom code (R ver 4.1.1) used to generate main plots of the manuscript with detailed information are available on GitHub: https://github.com/arianlundberg/BC-CCS

For manuscripts utilizing custom algorithms or software that are central to the research but not yet described in published literature, software must be made available to editors and reviewers. We strongly encourage code deposition in a community repository (e.g. GitHub). See the Nature Portfolio guidelines for submitting code & software for further information.

Data

Policy information about availability of data

All manuscripts must include a data availability statement. This statement should provide the following information, where applicable:

- Accession codes, unique identifiers, or web links for publicly available datasets
- A description of any restrictions on data availability
- For clinical datasets or third party data, please ensure that the statement adheres to our policy

All custom codes to generate the main figures of this manuscript are available on Github: https://github.com/arianlundberg/BC-CCS

Human research participants

Policy information about studies involving human research participants and Sex and Gender in Research.

Reporting on sex and gender	Analyses in this study were performed on the basis of BC-CCS tumour groupings regardless of sex
Population characteristics	TGCA populations characteristics have been previously extensively described in the publication "An Integrated TCGA Pan-Cancer Clinical Data Resource to Drive High-Quality Survival Outcome Analytics" published in Cell Volume 173, Issue 2, 5 April 2018, Pages 400-416.e11. GTEx samples and donor characteristics are also provided and visualised on the data website (https://gtexportal.org/home/tissueSummaryPage), and have previously extensively published.<br><br>Both datasets are taken from prominent public data repositories
Recruitment	A full statement on recruitment for donors is provided on the NHGRI website here: https://www.genome.gov/27549432/gtex-surgical-donors<br><br>For TCGA a full Human Subjects Protection and Data Access Policies statement is provided here by the NATIONAL CANCER INSTITUTE & NATIONAL HUMAN GENOME RESEARCH INSTITUTE: https://www.cancer.gov/about-nci/organization/ccg/research/structural-genomics/tcga/history/policies/tcga-human-subjects-data-policies.pdf
Ethics oversight	A set of policies developed by NCI and NHGRI to protect the privacy of participants donating specimens to TCGA. Included are TCGA's informed consent policy, data access policy and information about HIPAA Privacy Rule compliance. January, 2014<br>The Genotype-Tissue Expression (GTEx) project, supported by the National Institutes of Health (NIH) Common Fund, has collected and analyzed donated normal human biospecimens from deceased donors to form a resource database and tissue bank aimed at helping researchers better understand the relationship between human genetic variation and inherited susceptibility to disease. As part of the project, BBRB facilitated several studies looking at different aspects of the ethical, legal and social implications (ELSI) of GTEx. The ELSI studies included interviews with family decision makers who had been approached to donate their loved one's tissues, to assess the effectiveness of the consent process in informing the donor families of the potential impacts of participating in the study. Community Advisory Boards were also created to get community input into various issues including the development of improved consenting processes.

Note that full information on the approval of the study protocol must also be provided in the manuscript.

Field-specific reporting

Please select the one below that is the best fit for your research. If you are not sure, read the appropriate sections before making your selection.

☒ Life sciences ☐ Behavioural & social sciences ☐ Ecological, evolutionary & environmental sciences

For a reference copy of the document with all sections, see nature.com/documents/nr-reporting-summary-flat.pdf

Life sciences study design

All studies must disclose on these points even when the disclosure is negative.

Sample size	13,117 tumor samples
Data exclusions	From the original 19,131 samples from the Toil recompute project, we filtered out those that were not a part of GTEx and TCGA pan-cancer datasets (N = 734), that lacked mRNA-expression data (N = 92), that did not have representative tissue from the same site in both studies (N = 802) or where the normal tissue or cancer site contained fewer than ten samples (N = 4,043, Figure 1). Next, we assessed possible batch effects and the presence of outliers with PCA plots using RNA-seq data from the remaining 13,460 samples. Plotting on the basis of the most variable genes across all samples, PCA showed a long tail of samples stretching into the lower right quadrant. Closer inspection and annotation of these samples showed all were GTEx normal testicular tissue, indicating a potential study of origin batch effect and as such all normal and cancer testicular samples were removed from further analyses (N = 319). Similarly, after applying our Cell Cycle Score (CCS) signature to the remaining samples we noted a small number of outliers (N = 24) from mixed origin using PCA (Supplementary Figure 1C and D, circled in red) which were also removed from further analysis leaving 13,117 samples in total.

Replication	*Describe the measures taken to verify the reproducibility of the experimental findings. If all attempts at replication were successful, confirm this OR if there are any findings that were not replicated or cannot be reproduced, note this and describe why.*
Randomization	*Describe how samples/organisms/participants were allocated into experimental groups. If allocation was not random, describe how covariates were controlled OR if this is not relevant to your study, explain why.*
Blinding	*Describe whether the investigators were blinded to group allocation during data collection and/or analysis. If blinding was not possible, describe why OR explain why blinding was not relevant to your study.*

Reporting for specific materials, systems and methods

We require information from authors about some types of materials, experimental systems and methods used in many studies. Here, indicate whether each material, system or method listed is relevant to your study. If you are not sure if a list item applies to your research, read the appropriate section before selecting a response.

Materials & experimental systems

n/a	Involved in the study
☒	☐ Antibodies
☒	☐ Eukaryotic cell lines
☒	☐ Palaeontology and archaeology
☒	☐ Animals and other organisms
☒	☐ Clinical data
☒	☐ Dual use research of concern

Methods

n/a	Involved in the study
☒	☐ ChIP-seq
☒	☐ Flow cytometry
☒	☐ MRI-based neuroimaging
